# Detection and Structural Characterization of Nucleophiles Trapped Reactive Metabolites of Limonin Using Liquid Chromatography-Mass Spectrometry

**DOI:** 10.1155/2018/3797389

**Published:** 2018-04-17

**Authors:** Yujie Deng, Yudong Fu, Shumin Xu, Ping Wang, Nailong Yang, Chengqian Li, Qing Yu

**Affiliations:** ^1^Department of Endocrinology, The Affiliated Hospital of Qingdao University, 16 Jiangsu Road, Qingdao 266071, China; ^2^Department of Ophthalmology, The Affiliated Hospital of Qingdao University, 16 Jiangsu Road, Qingdao 266071, China

## Abstract

Limonin (LIM), a furan-containing limonoid, is one of the most abundant components of *Dictamnus dasycarpus* Turcz. Recent studies demonstrated that LIM has great potential for inhibiting the activity of drug-metabolizing enzymes. However, the mechanisms of LIM-induced enzyme inactivation processes remain unexplored. The main objective of this study was to identify the reactive metabolites of LIM using liquid chromatography-mass spectrometry. Three nucleophiles, glutathione (GSH), *N*-acetyl cysteine (NAC), and *N*-acetyl lysine (NAL), were used to trap the reactive metabolites of LIM in *in vitro* and *in vivo* models. Two different types of mass spectrometry, a hybrid quadrupole time-of-flight (Q-TOF) mass spectrometry and a LTQ velos Pro ion trap mass spectrometry, were employed to acquire structural information of nucleophile adducts of LIM. In total, six nucleophile adducts of LIM (M1–M6) with their isomers were identified; among them, M1 was a GSH and NAL conjugate of LIM, M2–M4 were glutathione adducts of LIM, M5 was a NAC and NAL conjugate of LIM, and M6 was a NAC adduct of LIM. Additionally, CYP3A4 was found to be the key enzyme responsible for the bioactivation of limonin. This metabolism study largely facilitates the understanding of mechanisms of limonin-induced enzyme inactivation processes.

## 1. Introduction

*Dictamnus dasycarpus* Turcz, known as Bai-Xian-Pi (BXP) in Chinese, belongs to the Rutaceae family. BXP has been widely used in Asian and European countries as an antipruritic, antidote, antibacterial, and anti-inflammatory agent. It is also used for the treatment of rubella, eczema, scabies, and jaundice. In addition, BXP displays diverse pharmacological properties, including antitumor, antiarrhythmic, antitinea, and smooth muscle-contraction activities [1]. Despite this, the safety of BXP has been questioned, and ingestion of BXP was reportedly associated with high incidence of liver injury. For instance, four cases of toxic hepatitis were reported in patients after taking a decoction made by boiling down the root of BXP [2]. In another clinical trial for the treatment of eczema, a standard mixture containing BXP was implicated in six of thirty-three cases of severe hepatitis [3].

Quinoline alkaloids, limonoids, sesquiterpenes, coumarins, flavonoids, and steroids have been explored as the major components of BXP [1, 4–8]. Limonoids have drawn much attention, and thus far a total of 25 limonoids have been isolated and characterized from BXP [1]; among them, Limonin (LIM) is one of the most abundant limonoids found in BXP [9]. Recent studies demonstrated that LIM has great potential for inhibiting the activity of drug-metabolizing enzymes and/or transporters such as CYP enzyme isomers and P-glycoprotein [10, 11]. As far as we know, modulation of activity of drug-metabolizing enzymes and/or transporters will result in the alteration of the clearance of exogenous toxins and affects the hepatic detoxification functions. However, the mechanisms of LIM-induced enzyme inactivation processes remain unknown.

**Scheme 1:**
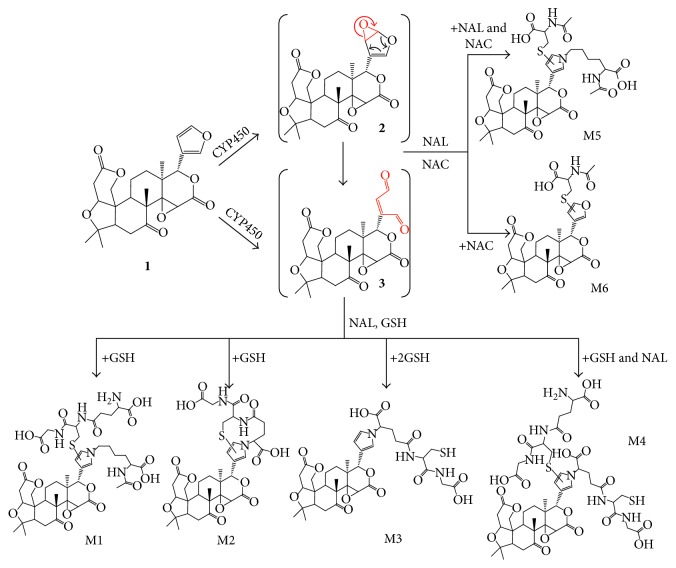
Microsomal metabolism of the furan ring of LIM and the proposed pathway for the formation of LIM-derived conjugates.

LIM is a furan-containing component. Many xenobiotics containing a furan unit are reported to be toxic and/or carcinogenic [12], such as furosemide [13], prazosin [14], teucrin A [15–17], 8-epidiosbulbin E [18–20], and diosbulbin B [21, 22]. The toxic effects elicited by these furans are suggested to be attributed to their *cis*-enedial oxidative intermediate [12]. We hypothesized that LIM is metabolized to a *cis*-enedial intermediate (**3**, Scheme 1), an electrophilic species, which may play a critical role in enzyme inactivation activities of LIM. In this study, we present the successful characterization of a *cis*-enedial intermediate of LIM and the identification of the cytochromes P450 (CYP450) enzymes responsible for the metabolic activation of LIM.

## 2. Materials and Methods

### 2.1. Chemicals and Materials

Mouse liver microsomes (MLMs), human liver microsomes (HLMs), recombinant human P450 enzymes, NADPH-regenerating system, glutathione (GSH), *N*-acetyl cysteine (NAC), and *N*-acetyl lysine (NAL) were purchased from BD Biosciences (Bedford, MA, USA). LIM (purity >98%) was obtained from Sigma-Aldrich (Sigma-Aldrich, St. Louis, MO, USA). Acetonitrile (ACN), methanol (MeOH), and formic acid (FA) of LC/MS grade were obtained from Fisher Scientific (Pittsburgh, PA, USA). Water was purified with a Milli-Q system (Millipore, Bedford, USA) and was passed through a 0.22 *μ*m membrane filter before use.

### 2.2. Animal Studies and Sample Collection

Experiments with mice were carried out according to the guidelines for Animal Experimentation of Qingdao University (Qingdao, China), and the protocol was approved by the Animal Ethics Committee of the institution. Female Kunming mice (20 ± 5 g) were obtained from the Experimental Animal Center of Qingdao University (Qingdao, China). Mice were housed 5 per cage and maintained in air-conditioned quarters with a room temperature of 20 ± 2°C, relative humidity of 50 ± 10%, and an alternating 12 h light/dark cycle and allowed to acclimate for at least 1 week prior to the start of the experiment. Mice were fed with standard chaw diet and water and were allowed to eat and drink *ad libitum*. LIM dissolved in dimethylsulfoxide (DMSO) was orally administered to mice at a signal dose of 5 mg/kg. Twenty-four-hour mouse urine and fecal samples were collected at room temperature by using metabolic cages. Blank urine and fecal samples were collected prior to the LIM treatment. These samples were stored under −80°C before analysis.

### 2.3. Sample Preparation

One hundred and fifty microliters of ACN was added to 50 *µ*L of urine sample, then vortexed for 3 min and centrifuged at 16,100 ×g for 10 min under 4°C. The supernatant was concentrated to dryness under a gentle stream of nitrogen gas at 45°C. The resulting residue was reconstituted with 200 *μ*L of ACN/water (50/50, v/v) containing 2% acetic acid, followed by centrifugation at 16,100 ×g for 10 min at 4°C. Ten *μ*L aliquot of the supernatant was injected into LC-MS/MS systems for analysis.

### 2.4. Microsomal Incubations

Liver microsomes with a final concentration at 0.5 mg/mL were incubated with LIM (30 *μ*M) for 60 min in the presence of GSH or NAC and NAL at a final concentration of 1.0 mM. The experimental incubation mixture consisted of 100 mM potassium phosphate buffer (pH 7.4), a prepared NADPH-regenerating system, and MLMs or HLMs. Stock solution of LIM was prepared in DMSO, and the final concentration of DMSO in the incubation did not exceed 1% (v/v). After preincubated at 37°C for 15 min in a water bath, the reactions were initiated by the addition of LIM and were incubated at 37°C for another 60 min. The reactions were terminated by the addition of an equal volume of ice-cold ACN containing 2% acetic acid. The mixture was vortexed and centrifuged at 16,100 ×g for 5 min. Aliquots of supernatants were stored at −20°C until analysis. Control incubations without NADPH-regenerating system, without substrate, or without liver microsomes were performed to ensure that the formation of the metabolites was microsome- and NADPH-dependent.

### 2.5. Chemical Synthesis

Five milligrams of LIM was completely dissolved in 400 *µ*l of acetone, then 50 *µ*L of saturated sodium bicarbonate solution, and 10 mg of Oxone were added successively to the resulting solution. The mixture was stirred for 15 min at room temperature, followed by addition of 50 mg of GSH or 22 mg of NAC, both GSH and NAC were dissolved in 500 *μ*L of saturated sodium bicarbonate solution. The mixture was stirred for 30 min and then centrifuged; the supernatants were harvested and evaporated to dryness under a stream of nitrogen gas at 45°C. The resulting residues were reconstituted with 500 *μ*L of pH 7.4 PBS buffer, then 5 mg of NAL was added and stirred for 30 min at 70°C, the reaction was cooled to room temperature and filtered through a 0.22 *µ*m member filter, and then analyzed by LC-MS/MS.

### 2.6. Recombinant Human P450 Enzyme Phenotyping

To determine the specific P450 enzymes involved in the formation of reactive metabolites of LIM, a total of 10 human recombinant P450s, including P450s 1A2, 2A6, 2B6, 2C8, 2C9, 2C19, 2D6, 2E1, 3A4, and 4A11 were screened. Conditions were equivalent to those of the microsomal incubations except that the microsomes were replaced by individual human recombinant P450 enzymes at a concentration of 25 pmol enzyme with a total volume of 200 *µ*L in each incubation. Experiments were performed in triplicate.

### 2.7. LC-MS/MS Method

All samples were analyzed on a Thermo-Finnigan spectra system consisting of an Ultimate 3000 degasser, an Ultimate 3000 RS pump, an Ultimate 3000 RS column compartment, and an LTQ Velos Pro ion trap mass spectrometer (Thermo Scientific, San Jose, CA) coupled with an electrospray ionization (ESI) interface. The ESI interface was operated in a positive ion polarity mode. The voltage on the ESI interface was maintained at approximately 4.3 kV and ESI capillary temperature was set at 300°C. Nitrogen gas was used as the sheath gas and auxiliary gas which was set at 35 and 10 units, respectively. The collision energy was set at 35 with isolation width of 2 Da for MS^2^. Chromatographic separation was performed on a Phenomenex Gemini C18 column (5 *μ*m, 3.0 mm i.d. × 150 mm; Torrance, CA, USA), and the column temperature was set at 35°C. The mobile phase was 5% aqueous MeOH with 0.1% formic acid (mobile phase A) and 95% aqueous MeOH with 0.1% formic acid (mobile phase B). The gradient was initiated at 90% A and held constant for 5 min, followed by linear increases in B to 25% from 5 to 10 min; to 60% from 10 to 30 min, to 100% from 30 to 40 min; and then held constant for 5 min. The column was then reequilibrated with 90% A for 5 min. The flow rate was set at 0.3 mL/min. The injection volume was 10 *μ*L for each sample. Data acquisition and analysis were performed using Xcalibur 2.2 version (Thermo Electron, San Jose, CA, USA).

Samples were also analyzed on a hybrid quadrupole time-of-flight (Q-TOF) mass spectrometer (Bruker micro Q-TOF, Bremen, Germany) with an electrospray ionization interface equipped with an Agilent 1200 series rapid resolution LC system. The mass spectrum data were acquired in the positive ion mode. The parameters of ESI-MS were set as follows: capillary voltage (−4.3 kV), the nebulizer gas pressure (1.2 bar), the dry gas flow rate (8.0 L/min), and temperature (220°C). The spectra were acquired at 2 s per spectrum in the range of *m*/*z* 100 to 1200. LC conditions were the same to those described above for the LTQ ion trap MS system. Data acquisition and analysis were performed using Bruker Daltonics data analysis 3.4 software.

## 3. Results

### 3.1. In Vitro Metabolic Activation of LIM

**Figure 1:**
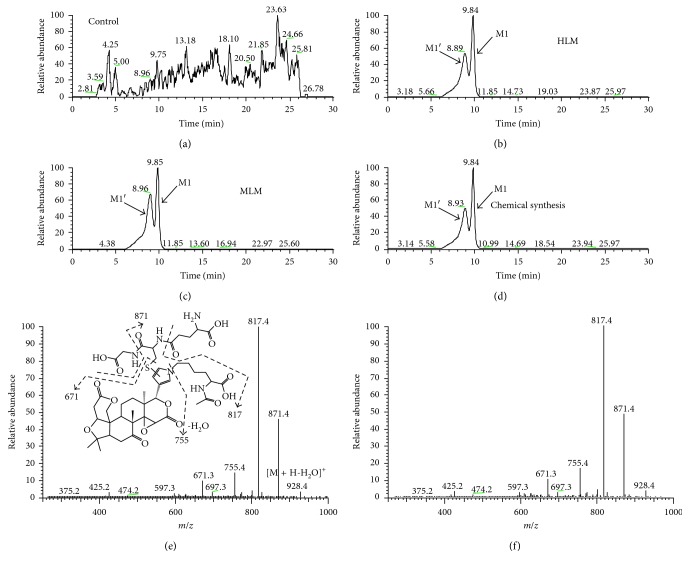
Extract ion (*m*/*z* 956 → 817) chromatograms obtained from LC-LTQ MS analysis of microsomal incubations containing LIM, GSH, NAL, and NADPH in the absence microsomes (a), or in presence of HLMs (b) or MLMs (c). (d) Extracted ion (*m*/*z* 956 → 817) chromatogram obtained from LC-LTQ MS analysis of synthetic M1 and M1′. (e) MS/MS spectrum of M1 generated in microsomal incubations (M1′ showed the same MS/MS spectrum). (f) MS/MS spectrum of synthetic M1 (synthetic M1′ showed the same MS/MS spectrum).

We proposed that the furan group of LIM played an important role in the LIM-induced inhibition of the activity of drug-metabolizing enzymes and/or transporters, and specifically this LIM is metabolized to the corresponding *cis*-enedial (Scheme 1, *cis*-enedial **3**), and the resulting electrophilic metabolites are responsible for the quench of many drug-metabolizing enzyme activities. LIM was incubated in MLMs or HLMs supplemented with GSH or NAC and NAL as trapping agents. The mixture was analyzed by a Thermo Scientific LTQ Velos ion trap mass spectrometer. Metabolites M1 and M1′ (retention times at 8.9 and 9.8 min, resp.) were detected (Figures 1(b) and 1(c)) in both HLMs and MLMs incubations by scanning of an ion pair of *m*/*z* 946 → 817, and M1 and M1′ showed identical mass fragmental patterns with indicative characteristic secondary ion signals associated the cleavage of the GSH moiety (Figure 1(e)). The product ions at *m*/*z* 928 were derived from the loss of one water molecule (−18 Da), and the product ions at *m*/*z* 871 and 817 were derived from the loss of glycine portion (−75 Da) and *γ*-glutamyl portion (−129 Da) from *m*/*z* 946, respectively. The mixture was also analyzed by LC/Q-TOF MS. M1 and M1′ showed their protonated molecule ion [M + H]^+^ at *m*/*z* 946.4512 and *m*/*z* 946.4513 in positive ion mode, respectively; both of them matches the elemental composition of C_44_H_60_N_5_O_16_S. No such conjugate was detected in the microsomal incubation system with the absence of NADPH (Figure 1(a)), indicating that the formation of M1 and M1′ was mediated by the microsomal metabolism. To further characterize these two metabolites, we oxidized LIM with Oxone in acetone, followed by the addition of GSH and NAL; two major products formed in the reaction showed the same chromatographic and mass spectrometric identities (Figures 1(d) and 1(f)) as that of the products (M1 and M1′) generated in the microsomal incubations. Unfortunately, we were unable to purify enough amount of the product for nuclear magnetic resonance (NMR) characterization.

**Figure 2:**
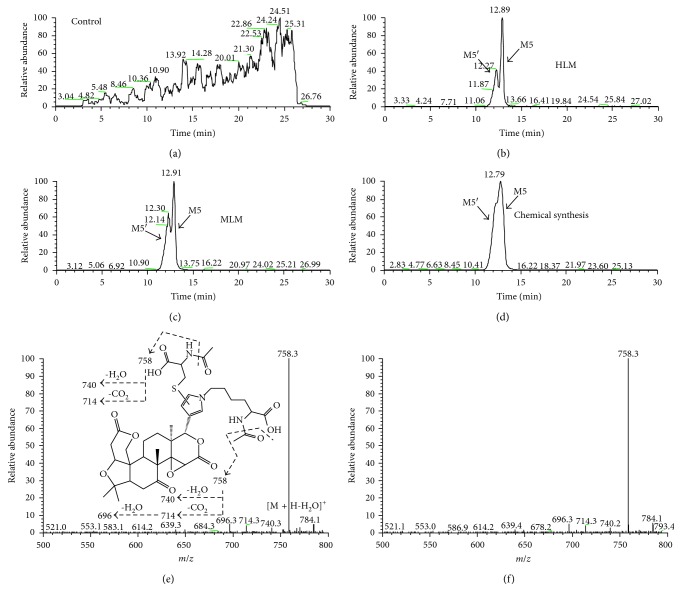
Extract ion (*m*/*z* 802 → 758) chromatograms obtained from LC-LTQ MS analysis of microsomal incubations containing LIM, GSH, NAL, and NADPH in the absence microsomes (a) or in presence of HLMs (b) or MLMs (c). (d) Extracted ion (*m*/*z* 802 → 758) chromatogram obtained from LC-LTQ MS analysis of synthetic M5 and M5′. (e) MS/MS spectrum of M5 generated in microsomal incubations (M5′ showed the same MS/MS spectrum). (f) MS/MS spectrum of synthetic M1 (synthetic M1′ showed the same MS/MS spectrum).

Interestingly, mono-GSH adducts of LIM were also observed by selected reaction monitoring (SRM) scanning with ion transition of *m*/*z* 758 → 683 and *m*/*z* 760 → 685 in the positive mode. Under the transition of *m*/*z* 758 → 683, metabolites M2 and M2′ (retention times at 12.9 and 12.2 min, resp.) were detected in both MLMs and HLMs incubation systems (Supplementary Figures 1(B) and 1(C)). The tandem mass spectrometry (MS/MS) spectrum of M2 and M2′ were identical, with one of the major fragmental ions at *m*/*z* 683, indicating the loss of glycine (−75 Da); the product ion at *m*/*z* 740 is postulated to arise from the elimination of H_2_O (Supplementary Figure 1(E)). Further analysis by LC/Q-TOF MS demonstrated that M2 and M2′ showed their protonated molecular ions at *m*/*z* 758.2351 and 758.2353, respectively, corresponding to the formula C_36_H_44_N_3_O_13_S. On the basis of the observed mass spectrometric data, we propose that M2 and M2′ are generated by intramolecular cyclization after GSH was conjugated to the *cis*-enedial intermediate which was derived from LIM. Under the transition of *m*/*z* 760 → 685, four metabolites (M3, M3′, M3″, and M3‴) with retention times at 12.9, 12.3, 11.4, and 10.6 min, respectively, were detected in HLMs incubation system by mass spectrometry (Supplementary Figure 1(B)) and two metabolites (M3 and M3′) with retention times of 12.9 and 12.3 min were observed in the MLMs incubation system; these metabolites showed identical mass fragmental patterns, provided major products ions at *m*/*z* 742, 685, 657, 614, and 552 (Supplementary Figure 2(E)). These metabolites were further analyzed by LC/Q-TOF MS, and they showed their protonated molecular ion [M + H]^+^ at around *m*/*z* 760.2152 in the positive ion mode, all of them in agreement with the elemental composition of C_36_H_46_N_3_O_13_S. We propose that these metabolites were isomers of a pyrrole-GSH conjugate (Scheme 1). To further characterize the metabolites detected under the transition of *m*/*z* 758 → 683 and *m*/*z* 760 → 685, we analyzed the mixture of the biomimetic oxidation of LIM described above. As expected, M2, M2′, M3, and M3′ were all detected, based on their retention times, molecular ions, and MS/MS spectra (Supplementary Figures 1(D) and 1(E); Figures 2(d) and 2(e)).

Metabolites M4 and M4′, with retention times at 9.6 and 8.5 min, were observed by scanning of an ion pair of *m*/*z* 1065 → 936 in the positive ion mode (Supplementary Figure 3(B)). The MS/MS spectrum of M4 and M4′ were identical which showed the major fragment ions associated with fragmentation of the GSH moiety (Supplementary Figure 3(E)). The product ions at *m*/*z* 990 and 936 were derived from the loss of glycinyl moiety (−75 Da) and *γ*-glutamyl moiety (−129 Da) from *m*/*z* 1065, respectively. This indicates the participation of GSH in the formation of M4 and M4′. Further analysis by LC-Q/TOF MS showed its molecular ion at *m*/*z* 1065.3431. The protonated molecular ions observed were consistent with the molecular mass corresponding to the elemental composition of C_46_H_61_N_6_O_19_S_2_, suggesting that M4 and M4′ are derived from two molecules of GSH, which are LIM-derived di-GSH conjugates (Scheme 1).

In a parallel incubation, NAC in place of GSH was used to trap the LIM-derived *cis*-enedial intermediate. No such adducts like M1, M2, M3, and M4, which were found in GSH-fortified microsomal incubations, were observed in the NAC supplied microsomal incubation systems. Instead, we detected two metabolites (M5 and M6) most likely associated with NAC/NAL. Under the ion transition of *m*/*z* 802 → 758, M5 and M5′ were detected in both HLM and MLM incubation systems with retention times at 12.9 and 12.3 min (Figures 2(b) and 2(c)), respectively. These metabolites were further analyzed by LC/Q-TOF MS; M5 and M5′ showed their protonated molecule ion [M + H]^+^ at *m*/*z* 802.3217 in positive ion mode, which matches the elemental composition of C_39_H_52_N_3_O_13_S. The MS/MS spectrum of M5 and M5′ identically showed the indicative characteristic neutral loss of one macular of water (−18 Da) and successive losses of 44 Da and/or 18 Da. The product ions at *m*/*z* 784, 758, 740, 714, and 696 were assigned to [M + H-H_2_O]^+^, [M + H-CO_2_]^+^, [M + H-CO_2_-H_2_O]^+^, [M + H-2CO_2_]^+^, and [M + H-2CO_2_-H_2_O]^+^, respectively (Figure 2(e)). The formation of M5 and M5′ were also found to be NADPH dependent (data not shown). To further characterize M5 and M5′, LIM was oxidized with Oxone in acetone, follow by reaction with NAC and NAL. Two major products formed in the reaction showed the same chromatographic and mass spectrometric identities as that of the metabolites M5 and M5′ generated in the microsomal incubation systems (Figures 2(d) and 2(f)). Besides M5 and M5′, a mono-NAC adduct of LIM (M6) was observed under ion transition of *m*/*z* 632 → 614 in positive ion mode. Fragmental ion generated from a natural loss of one macular of water (−18 Da), *m*/*z* 614, was identified as the major fragmental ion; to further characterize this metabolite, we analyzed the mixture of the biomimetic oxidation of LIM described above. As expected, a product formed in the reaction showed the same chromatographic and mass spectrometric identities as that of the product (M6) generated in the microsomal incubations (Supplementary Figures 4(D) and 4(F)), and this metabolite was tentatively identified as mono-NAC adduct of LIM.

### 3.2. Metabolic Activation of LIM in Mice

**Figure 3:**
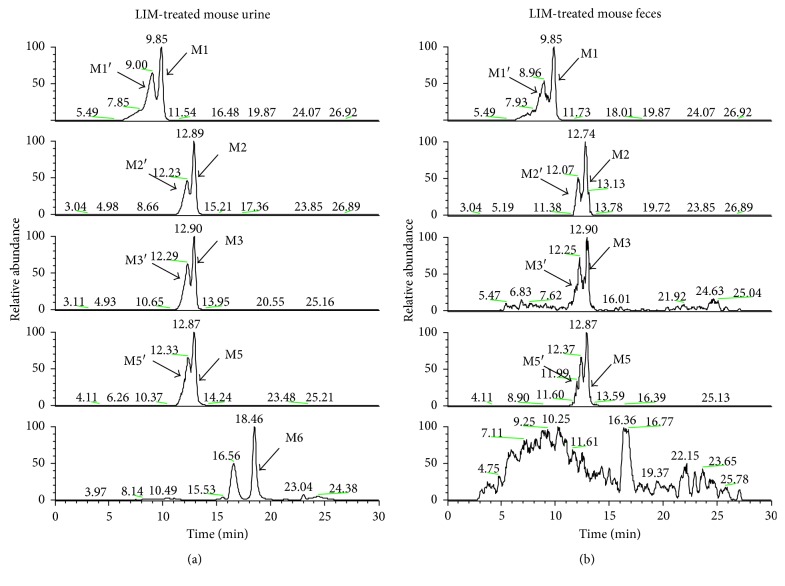
Extracted ion (*m*/*z* 946 → 817, *m*/*z* 758 → 683, *m*/*z* 760 → 685, *m*/*z* 802 → 758, and *m*/*z* 632 → 614, represent M1, M2, M3, M5, and M6, resp.) chromatograms obtained from LC-LTQ MS analysis of urine (a), and feces (b) of mice after the treatment of LIM.

To investigate the bioactivation of LIM *in vivo*, urine, and fecal samples collected from LIM treated mice were monitored by a designed selected reaction monitoring (SRM) template using LC-MS/MS. One GSH and NAL adduct (M1), two mono-GSH adducts (M2 and M3), one NAC and NAL adduct (M5), and one mono-NAC adduct (M6) were found in the urinary samples collected from the mice after given LIM (Figure 3(a)), except M6, these metabolites were also detected from the LIM-treated mouse fecal samples, while no such metabolites were observed from the blank mouse urine and fecal samples.

### 3.3. P450 Enzymes Responsible for the Bioactivation of LIM

**Figure 4:**
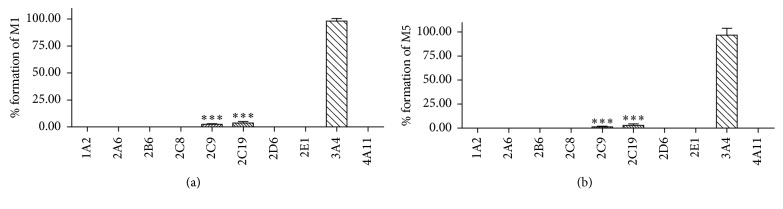
Rates of the M1 (A) and M5 (B) formation in incubations of LIM with recombinant human P450 enzymes.

In order to identify the P450 enzymes involved in the bioactivation, LIM was incubated with individual recombinant human P450s, including P450s 1A2, 2A6, 2B6, 2C8, 2C9, 2C19, 2D6, 2E1, 3A4, and 4A11, followed by monitoring the formation of M1 and M5. The bioactivation activities of each P450s were examined, and P450 2C9, 2C19, and 3A4 displayed the metabolic activity, with 3A4 as the most potent one (Figures 4(a) and 4(b)). These results clearly confirmed that P450 2C9, 2C19, and 3A4 were the primary enzymes that were involved in the bioactivation of LIM.

## 4. Conclusions

The citrus bitter principle limonin was first isolated in 1841, but intensive investigation of its bioactivity did not commence until the last decade. Metabolism study of LIM is still very limited, possibly due to little attention being paid to its toxic effects. The unexpected inhibition activity of LIM towards the drug-metabolizing enzymes and/or transporters led us to investigate the metabolic activation of LIM. We hypothesized that LIM is metabolized to an electrophilic species, a *cis*-enedial intermediate, then reactive with the nucleophilic side residues of proteins (thiol, alcohol, phenol, carboxyl, and amine), and led to the inactivation of protein functions.

In our current study, GSH which contains two nucleophilic functional groups, including a sulfhydryl (the cysteine residue) and an amino (the glycine residue) group, NAC and NAL each contains a nucleophilic amino group in its side chain, were chosen as the trapping reagents to trap the potential LIM-derived *cis*-enedial intermediate in mouse and human liver microsomal incubation systems after exposure to LIM. LC-MS/MS analysis showed a total of six LIM-derived conjugates (M1–M6) with their isomers in the microsomal incubations. On the basis of the molecular ions obtained from high-resolution mass spectrometry, six LIM-derived pyrrole derivatives are postulated to be formed in the microsomal incubation systems. Mechanistically, a sulfhydryl group and a primary amine group participate in the formation of the pyrrole derivatives. The pyrrole production possibly starts with the reaction of the *cis*-enedial intermediate with the sulfhydryl residue of GSH or NAC by Michael addition to form the corresponding GSH or NAC conjugate, which subsequently reacts with the amino residue of NAL to form a Schiffs base followed by intramolecular cyclization and dehydration to produce the pyrrole derivative. For the formation of M1, the sulfhydryl and amino groups came from GSH and NAL, respectively. For the generation of M2 and M3, the nucleophilic group was coming from GSH. M4 is a di-GSH conjugate, and the sulfhydryl and amino groups were acquired from the respective GSH molecules. Unlike the formation of M1-M4, for the generation M5 and M6, the sulfhydryl came from NAC, and the amino group was acquired from NAL. The *in vitro* findings for M1–M6 provided important evidence for the formation of *cis*-enedial intermediate in microsomal reactions. M1, M2, M3, M5, and M6, which were found in microsomal incubations, were also detected in the urine samples of mice given LIM (Figure 3); M1, M2, M3, and M5 were detected in LIM treated mouse fecal samples as well (Figure 3). It appears that the primary LIM-derived GSH, NAC, and/or NAL conjugates are excreted mainly through the urine with relatively trace amount they excreted through feces. However, M4, which identified as a LIM-derived di-GSH conjugate, was not detected from the mouse urine and fecal samples after LIM treatment. Nevertheless, the observation of the GSH or NAC and NAL conjugates in urine and feces infers the metabolism of LIM to the *cis*-enedial intermediate *in vivo*. In addition, bioactivation studies with individual recombinant enzymes demonstrated that P450 2C9, 2C19, and 3A4 are enzymes responsible for the bioactivation of LIM (Figure 4(a)), with P450 3A4 as the most potent one. These findings facilitate our ongoing investigation of the biochemical mechanisms of LIM-induced enzyme inactivation.

In summary, the metabolic generation of the *cis*-enedial intermediate from LIM was evident in both *in vitro* and *in vivo* systems. The condensation reaction of the electrophilic intermediate with GSH or NAC and NAL gave six GSH or NAC and NAL conjugates derived from the *cis*-enedial intermediate of LIM. P450 3A4 was identified as the dominant participant in the catalysis leading to the formation of the reactive *cis*-enedial intermediate. The metabolite identification work performed herein enables us to better understand the mechanisms of LIM-induced enzyme inactivation processes.

## Supplementary Materials

Supplementary 1 Figure 1: extract ion (*m*/*z* 758 → 683) chromatograms obtained from LC-LTQ MS analysis of microsomal incubations containing LIM, GSH, NAL, and NADPH in the absence microsomes (A), or in presence of HLMs (B) or MLMs (C). (D) Extracted ion (*m*/*z* 802 → 758) chromatogram obtained from LC-LTQ MS analysis of synthetic M2 and M2′. (E) MS/MS spectrum of M2 generated in microsomal incubations (M2′ showed the same MS/MS spectrum). (F) MS/MS spectrum of synthetic M2 (synthetic M2′ showed the same MS/MS spectrum).

Supplementary 2 Figure 2: extract ion (*m*/*z* 760 → 685) chromatograms obtained from LC-LTQ MS analysis of microsomal incubations containing LIM, GSH, NAL, and NADPH in the absence microsomes (A), or in presence of HLMs (B) or MLMs (C). (D) Extracted ion (*m*/*z* 760 → 685) chromatogram obtained from LC-LTQ MS analysis of synthetic M3 and M3′. (E) MS/MS spectrum of M3 generated in microsomal incubations (M3′ showed the same MS/MS spectrum). (F) MS/MS spectrum of synthetic M3 (synthetic M3′ showed the same MS/MS spectrum).

Supplementary 3 Figure 3: extract ion (*m*/*z* 1065 → 936) chromatograms obtained from LC-LTQ MS analysis of microsomal incubations containing LIM, GSH, NAL, and NADPH in the absence microsomes (A), or in presence of HLMs (B) or MLMs (C). (D) Extracted ion (*m*/*z* 1065 → 936) chromatogram obtained from LC-LTQ MS analysis of synthetic M4 and M4′. (E) MS/MS spectrum of M3 generated in microsomal incubations (M4′ showed the same MS/MS spectrum). (F) MS/MS spectrum of synthetic M4 (synthetic M4′ showed the same MS/MS spectrum).

Supplementary 4 Figure 4: extract ion (*m*/*z* 632 → 614) chromatograms obtained from LC-LTQ MS analysis of microsomal incubations containing LIM, NAC, NAL, and NADPH in the absence microsomes (A), or in presence of HLMs (B) or MLMs (C). (D) Extracted ion (*m*/*z* 632 → 614) chromatogram obtained from LC-LTQ MS analysis of synthetic M6. (E) MS/MS spectrum of M6 generated in microsomal incubations. (F) MS/MS spectrum of synthetic M6.
