# Supplementary material for: Detection and Structural Characterization of Nucleophiles Trapped Reactive Metabolites of Limonin Using Liquid Chromatography-Mass Spectrometry
Source: J Anal Methods Chem. 2018 Apr 17;2018:3797389. doi: 10.1155/2018/3797389 (PMC5932435; doi:10.1155/2018/3797389)
Supplement: Supplementary 1 — Figure 1: extract ion (m/z 758 → 683) chromatograms obtained from LC-LTQ MS analysis of microsomal incubations containing LIM, GSH, NAL, and NADPH in the absence microsomes (A), or in presence of HLMs (B) or MLMs (C). (D) Extracted ion (m/z 802 → 758) chromatogram obtained from LC-LTQ MS analysis of synthetic M2 and M2′. (E) MS/MS spectrum of M2 generated in microsomal incubations (M2′ showed the same MS/MS spectrum). (F) MS/MS spectrum of synthetic M2 (synthetic M2′ showed the same MS/MS spectrum). [file 3797389.f1.pptx]

## Slide 1
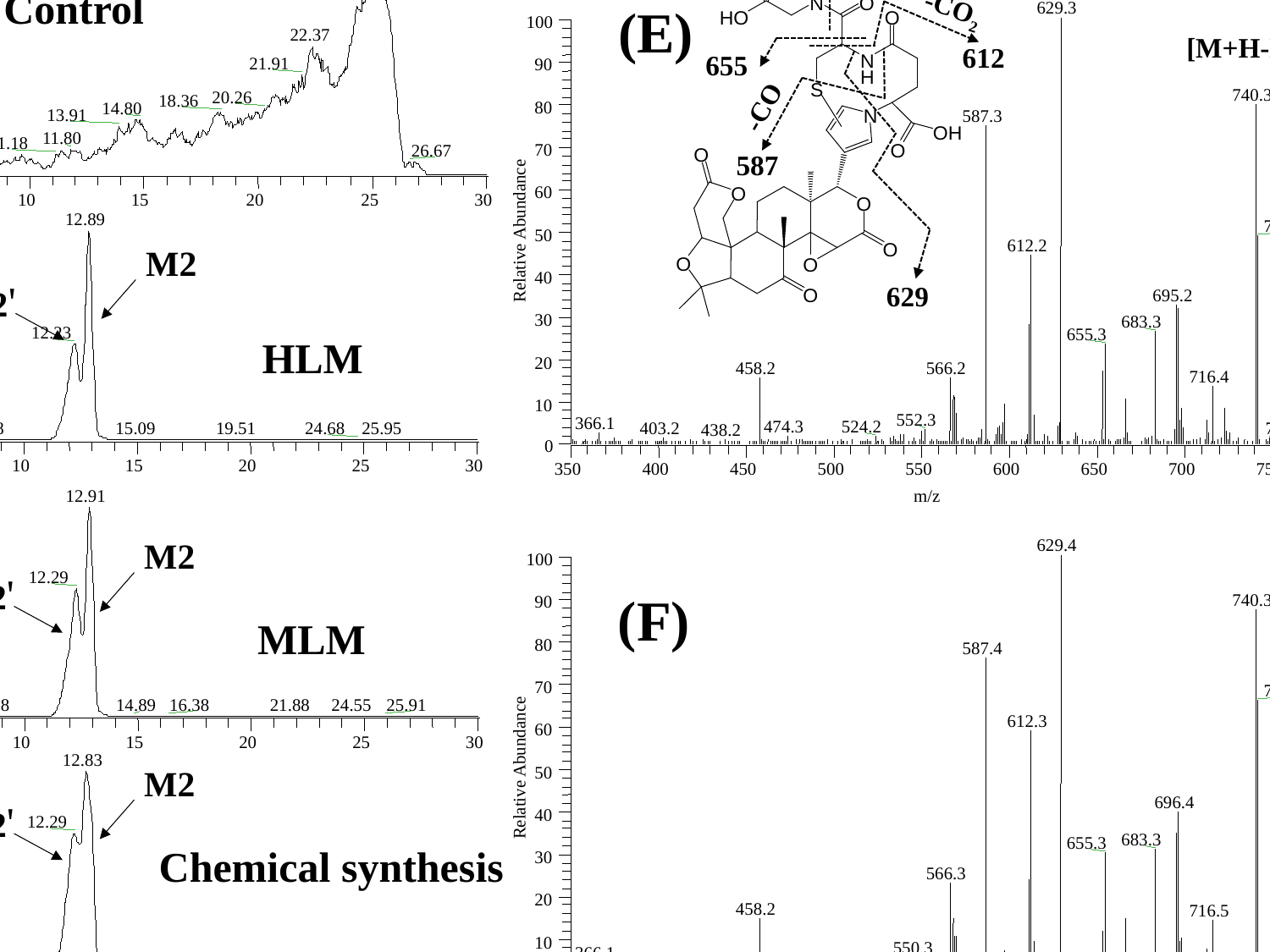

25.15
100
24.73
80
22.37
60
21.91
Relative Abundance
40
20.26
18.36
14.80
13.91
3.16
20
11.80
3.95
11.18
26.67
0
0
5
10
15
20
25
30
(A)
Control
12.89
100
80
60
12.23
Relative Abundance
40
20
1.84
4.07
5.64
8.58
15.09
19.51
24.68
25.95
0
0
5
10
15
20
25
30
(B)
HLM
12.91
100
80
12.29
60
Relative Abundance
40
20
3.20
5.18
8.78
14.89
16.38
21.88
24.55
25.91
0
0
5
10
15
20
25
30
(C)
MLM
12.83
100
80
12.29
60
Relative Abundance
40
20
5.22
8.70
15.02
16.51
22.05
24.59
25.83
0
0
5
10
15
20
25
30
Time (min)
(D)
Chemical synthesis
683
-CO2
612
655
-CO
587
629
100
90
80
70
60
Relative Abundance
50
40
30
20
10
0
350
400
450
500
550
600
650
700
750
m/z
629.3
740.3
587.3
741.3
612.2
695.2
683.3
655.3
458.2
566.2
716.4
552.3
366.1
474.3
524.2
403.2
748.7
438.2
(E)
[M+H-H2O]+
M2
M2'
100
90
80
70
60
Relative Abundance
50
40
30
20
10
0
350
400
450
500
550
600
650
700
750
m/z
629.4
740.3
587.4
741.3
612.3
696.4
683.3
655.3
566.3
458.2
716.5
550.3
366.1
742.2
403.3
524.4
482.4
446.6
M2
M2'
(F)
M2
M2'
Supplemental Figure 1. Extract ion (m/z 758 → 683) chromatograms obtained from LC-LTQ MS analysis of microsomal incubations containing LIM, GSH, NAL, and NADPH in the absence microsomes (A), or in presence of HLMs (B) or MLMs (C). (D) Extracted ion (m/z 802 → 758) chromatogram obtained from LC-LTQ MS analysis of synthetic M2 and M2'. (E) MS/MS spectrum of M2 generated in microsomal incubations (M2' showed the same MS/MS spectrum). (F) MS/MS spectrum of synthetic M2 (synthetic M2' showed the same MS/MS spectrum).
