# Supplementary material for: Detection and Structural Characterization of Nucleophiles Trapped Reactive Metabolites of Limonin Using Liquid Chromatography-Mass Spectrometry
Source: J Anal Methods Chem. 2018 Apr 17;2018:3797389. doi: 10.1155/2018/3797389 (PMC5932435; doi:10.1155/2018/3797389)
Supplement: Supplementary 2 — Figure 2: extract ion (m/z 760 → 685) chromatograms obtained from LC-LTQ MS analysis of microsomal incubations containing LIM, GSH, NAL, and NADPH in the absence microsomes (A), or in presence of HLMs (B) or MLMs (C). (D) Extracted ion (m/z 760 → 685) chromatogram obtained from LC-LTQ MS analysis of synthetic M3 and M3′. (E) MS/MS spectrum of M3 generated in microsomal incubations (M3′ showed the same MS/MS spectrum). (F) MS/MS spectrum of synthetic M3 (synthetic M3′ showed the same MS/MS spectrum). [file 3797389.f2.pptx]

## Slide 1
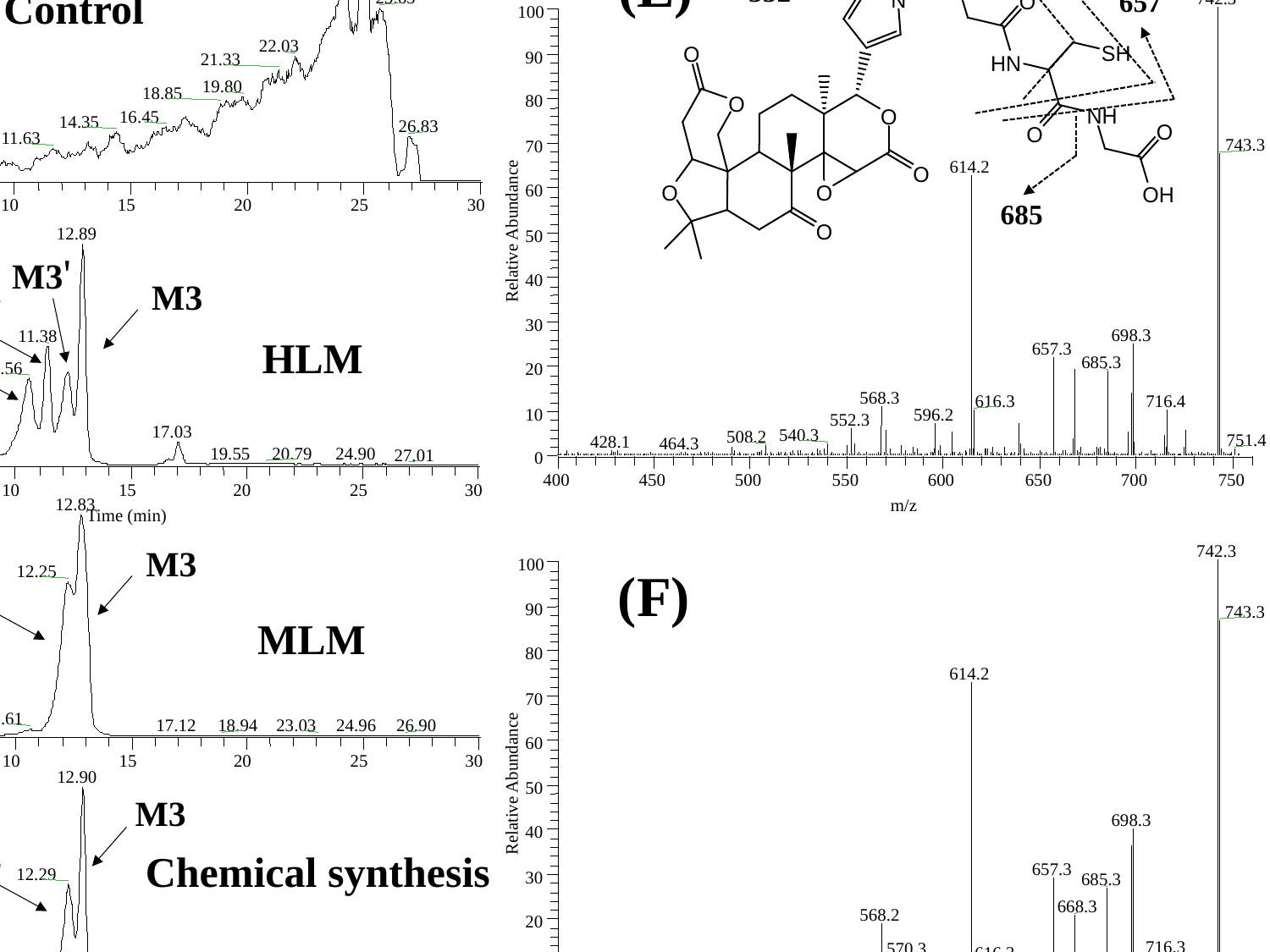

614
[M+H-H2O]+
614
100
90
80
70
60
Relative Abundance
50
40
30
20
10
0
400
450
500
550
600
650
700
750
m/z
742.3
743.3
614.2
698.3
657.3
685.3
568.3
616.3
716.4
596.2
552.3
540.3
508.2
751.4
428.1
464.3
(E)
552
657
685
100
90
80
70
60
Relative Abundance
50
40
30
20
10
0
400
450
500
550
600
650
700
750
m/z
742.3
743.3
614.2
698.3
657.3
685.3
668.3
568.2
716.3
570.3
616.3
552.3
540.2
744.0
508.2
405.3
464.3
(F)
24.88
100
25.19
24.03
25.65
80
22.03
60
21.33
Relative Abundance
19.80
18.85
40
16.45
14.35
26.83
11.63
20
9.19
3.53
6.96
0
0
5
10
15
20
25
30
(A)
Control
12.89
100
80
60
11.38
Relative Abundance
10.56
40
20
17.03
19.55
20.79
24.90
3.69
5.64
8.16
27.01
0
0
5
10
15
20
25
30
Time (min)
(B)
M3'
M3
M3''
HLM
M3'''
12.83
100
80
12.25
60
Relative Abundance
40
20
10.61
5.10
9.01
17.12
18.94
23.03
24.96
26.90
0
0
5
10
15
20
25
30
(C)
M3
M3'
MLM
SM:
12.90
100
80
12.29
60
Relative Abundance
40
20
10.65
3.86
5.26
9.01
14.39
17.08
23.23
25.08
27.07
0
0
5
10
15
20
25
30
Time (min)
(D)
M3
Chemical synthesis
M3'
Supplemental Figure 2. Extract ion (m/z 760 → 685) chromatograms obtained from LC-LTQ MS analysis of microsomal incubations containing LIM, GSH, NAL, and NADPH in the absence microsomes (A), or in presence of HLMs (B) or MLMs (C). (D) Extracted ion (m/z 760 → 685) chromatogram obtained from LC-LTQ MS analysis of synthetic M3 and M3'. (E) MS/MS spectrum of M3 generated in microsomal incubations (M3' showed the same MS/MS spectrum). (F) MS/MS spectrum of synthetic M3 (synthetic M3' showed the same MS/MS spectrum).
