# Supplementary material for: Detection and Structural Characterization of Nucleophiles Trapped Reactive Metabolites of Limonin Using Liquid Chromatography-Mass Spectrometry
Source: J Anal Methods Chem. 2018 Apr 17;2018:3797389. doi: 10.1155/2018/3797389 (PMC5932435; doi:10.1155/2018/3797389)
Supplement: Supplementary 3 — Figure 3: extract ion (m/z 1065 → 936) chromatograms obtained from LC-LTQ MS analysis of microsomal incubations containing LIM, GSH, NAL, and NADPH in the absence microsomes (A), or in presence of HLMs (B) or MLMs (C). (D) Extracted ion (m/z 1065 → 936) chromatogram obtained from LC-LTQ MS analysis of synthetic M4 and M4′. (E) MS/MS spectrum of M3 generated in microsomal incubations (M4′ showed the same MS/MS spectrum). (F) MS/MS spectrum of synthetic M4 (synthetic M4′ showed the same MS/MS spectrum). [file 3797389.f3.pptx]

## Slide 1
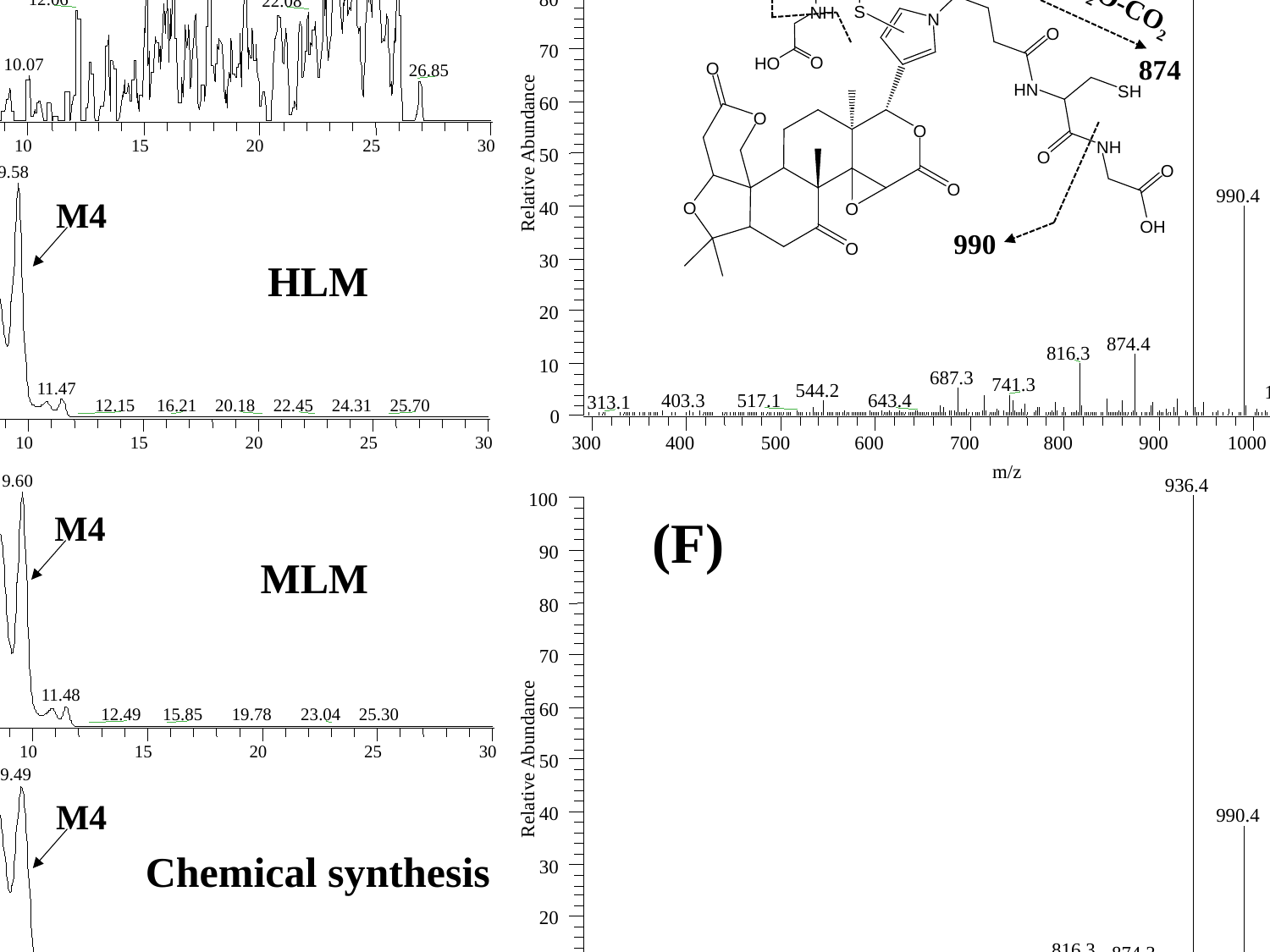

100
90
80
70
60
Relative Abundance
50
40
30
20
10
0
300
400
500
600
700
800
900
1000
1100
1200
m/z
936.4
990.4
874.4
816.3
687.3
741.3
544.2
1047.5
403.3
517.1
643.4
313.1
1128.4
25.04
100
25.16
24.04
80
16.26
25.94
17.83
15.06
22.87
60
12.06
22.08
Relative Abundance
40
10.07
7.67
26.85
20
5.81
4.70
2.92
0
0
5
10
15
20
25
30
(A)
Control
(E)
990
936
-H2O-CO2
874
(B)
9.58
100
8.46
80
60
Relative Abundance
40
3.91
20
11.47
12.15
16.21
20.18
22.45
24.31
25.70
0
0
5
10
15
20
25
30
M4
M4'
990
HLM
[M+H-H2O]+
100
90
80
70
60
Relative Abundance
50
40
30
20
10
0
300
400
500
600
700
800
900
1000
1100
1200
m/z
936.4
990.4
816.3
874.3
687.3
741.3
544.1
1047.6
375.2
653.3
442.1
1092.3
1195.1
9.60
100
8.58
80
60
Relative Abundance
40
3.95
20
11.48
3.04
12.49
15.85
19.78
23.04
25.30
0
0
5
10
15
20
25
30
(C)
M4
(F)
M4'
MLM
9.49
100
8.38
80
60
Relative Abundance
40
3.83
20
10.65
12.74
16.00
19.48
22.41
25.25
26.69
0
0
5
10
15
20
25
30
Time (min)
(D)
M4
M4'
Chemical synthesis
Supplemental Figure 3. Extract ion (m/z 1065 → 936) chromatograms obtained from LC-LTQ MS analysis of microsomal incubations containing LIM, GSH, NAL, and NADPH in the absence microsomes (A), or in presence of HLMs (B) or MLMs (C). (D) Extracted ion (m/z 1065 → 936) chromatogram obtained from LC-LTQ MS analysis of synthetic M4 and M4'. (E) MS/MS spectrum of M3 generated in microsomal incubations (M4' showed the same MS/MS spectrum). (F) MS/MS spectrum of synthetic M4 (synthetic M4' showed the same MS/MS spectrum).
