# Supplementary material for: Detection and Structural Characterization of Nucleophiles Trapped Reactive Metabolites of Limonin Using Liquid Chromatography-Mass Spectrometry
Source: J Anal Methods Chem. 2018 Apr 17;2018:3797389. doi: 10.1155/2018/3797389 (PMC5932435; doi:10.1155/2018/3797389)
Supplement: Supplementary 4 — Figure 4: extract ion (m/z 632 → 614) chromatograms obtained from LC-LTQ MS analysis of microsomal incubations containing LIM, NAC, NAL, and NADPH in the absence microsomes (A), or in presence of HLMs (B) or MLMs (C). (D) Extracted ion (m/z 632 → 614) chromatogram obtained from LC-LTQ MS analysis of synthetic M6. (E) MS/MS spectrum of M6 generated in microsomal incubations. (F) MS/MS spectrum of synthetic M6. [file 3797389.f4.pptx]

## Slide 1
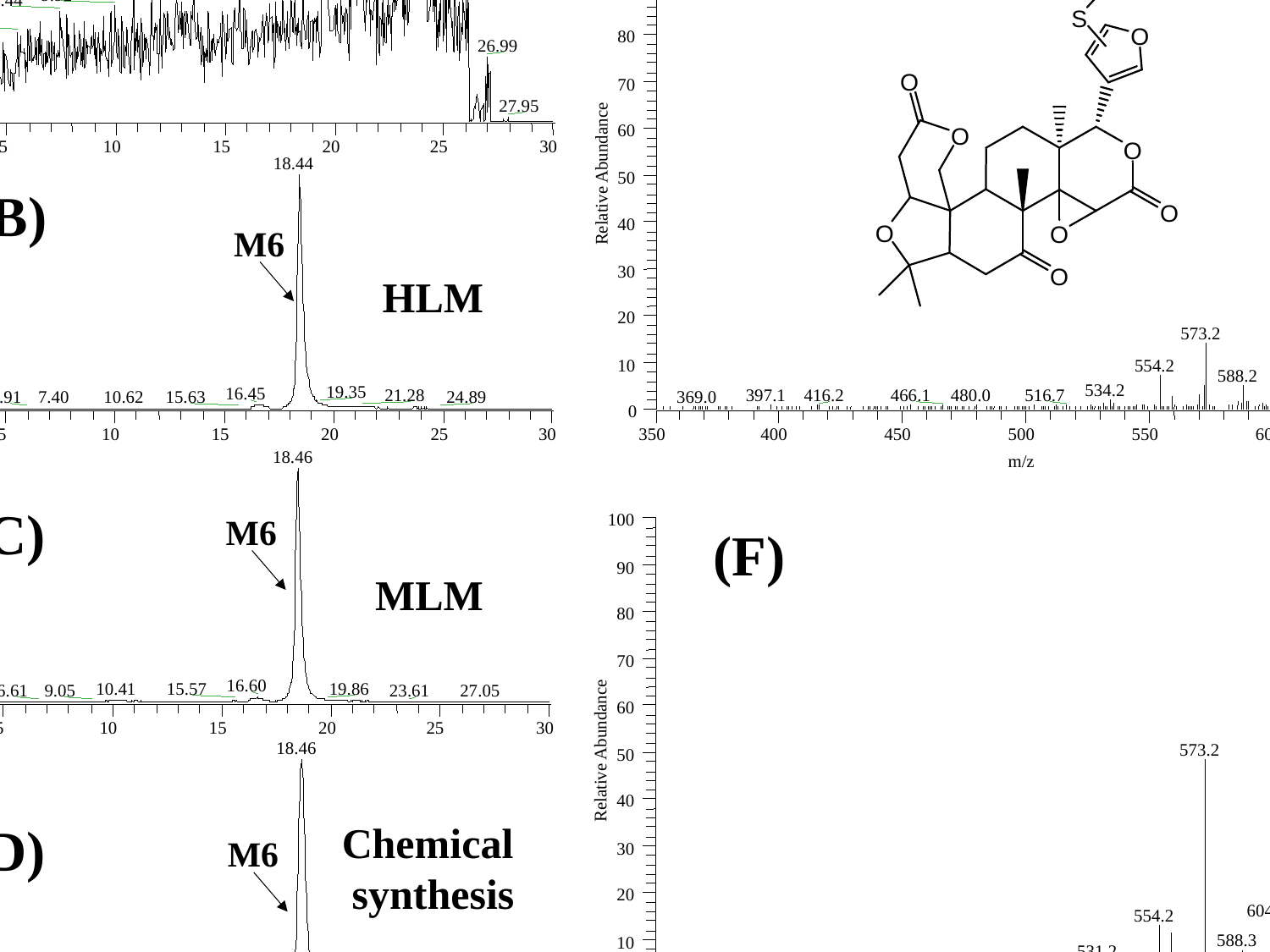

(A)
Control
588
23.64
19.53
100
80
24.06
25.49
15.65
13.52
60
10.79
9.92
7.44
Relative Abundance
5.50
40
26.99
4.59
20
1.07
27.95
0
0
5
10
15
20
25
30
[M+H-H2O]+
573
(E)
614.2
100
90
80
70
60
Relative Abundance
50
40
30
20
10
0
350
400
450
500
550
600
650
m/z
573.2
554.2
588.2
617.3
534.2
397.1
416.2
466.1
480.0
516.7
369.0
18.44
100
80
60
Relative Abundance
40
20
19.35
16.45
21.28
3.22
5.91
7.40
10.62
15.63
24.89
0
0
5
10
15
20
25
30
(B)
M6
HLM
18.46
100
80
60
Relative Abundance
40
20
16.60
10.41
15.57
19.86
4.46
6.61
9.05
23.61
27.05
0
0
5
10
15
20
25
30
100
90
80
70
60
Relative Abundance
50
40
30
20
10
0
350
400
450
500
550
600
650
m/z
614.2
573.2
604.2
554.2
588.3
617.1
531.2
466.0
517.3
375.2
415.3
438.0
637.5
(C)
M6
(F)
MLM
18.46
100
80
60
Relative Abundance
40
20
16.56
9.13
10.41
15.57
21.18
4.13
6.53
23.73
25.78
0
0
5
10
15
20
25
30
Time (min)
(D)
Chemical
synthesis
M6
Supplemental Figure 4. Extract ion (m/z 632 → 614) chromatograms obtained from LC-LTQ MS analysis of microsomal incubations containing LIM, NAC, NAL, and NADPH in the absence microsomes (A), or in presence of HLMs (B) or MLMs (C). (D) Extracted ion (m/z 632 → 614) chromatogram obtained from LC-LTQ MS analysis of synthetic M6. (E) MS/MS spectrum of M6 generated in microsomal incubations. (F) MS/MS spectrum of synthetic M6.
